# Synergistic effects and mechanisms of impressic acid or acankoreanogein in combination with docetaxel on prostate cancer†

**DOI:** 10.1039/c7ra11647k

**Published:** 2018-01-12

**Authors:** Sen Jiang, Kun Zhang, Yan He, Xuetao Xu, Dongli Li, Shupeng Cheng, Xi Zheng

**Affiliations:** Laboratory of Natural Medicinal Chemistry & Green Chemistry, Guangdong University of Technology Guangzhou P. R. China kzhang@gdut.edu.cn ahjxjiangsen@163.com hxg_129@126.com csp201014@126.com +86 750 3319012 +86 750 3296601; School of Chemical & Environmental Engineering, Wuyi University Jiangmen P. R. China wyuchemxxt@126.com wyuchemldl@126.com; International Healthcare Innovation Institute Jiangmen P. R. China; Susan Lehman Cullman Laboratory for Cancer Research, Department of Chemical Biology, Ernest Mario School of Pharmacy, Rutgers, The State University of New Jersey Piscataway NJ USA xizheng@pharmacy.rutgers.edu

## Abstract

Prostate cancer (PCa) is a common cancer among males and a leading cause of cancer deaths. Docetaxel (DOC) was recommended in guidelines as the first first-line drug of PCa; however, treatment with high doses of DOC ultimately results in resistance. This study examined the proliferation, viability, and apoptosis of VCaP cells evaluated by the MTT assay, trypan blue exclusion assay, and morphological assessments to investigate the effects and mechanisms of action by impressic acid (E12-1) or acankoreanogein (E13-1), isolated from *Acanthopanax trifoliatus* (L.) Merr., in combination with DOC in VCaP PCa cells. The research, which also contained cell migration, was examined under a light microscope. Nuclear factor kappa-light-chain-enhancer of activated B cells (NF-κB) activity was assessed by the luciferase reporter assay. Finally, the expression of B-cell lymphoma 2 (Bcl-2), NF-κB, phosphorylated Akt (p-Akt), phosphorylated signal transducer and activator of transcription 3 (p-Stat 3), phosphorylated c-Jun N-terminal kinase (p-JNK), and extracellular signal-related protein kinases 1 and 2 in VCaP cells was evaluated by western blotting. The result is combination of DOC with E12-1 or E13-1 which synergistically inhibited growth, induced apoptosis, and reduced migration of VCaP cells compared with treatment with DOC, E12-1, or E13-1 alone. The potential molecular mechanisms were related to significant decreases in the expression of NF-κB, Bcl-2, p-Stat 3, p-JNK, and p-Akt in VCaP cells. DOC combined with E12-1 or E13-1 may be an effective approach for inhibiting the growth and apoptosis of PCa cells, thus making it possible to reduce the dose of DOC in patients with PCa who experience systemic toxicity.

## Abstract 2

Prostate cancer (PCa) is a common cancer among males and a leading cause of cancer deaths.

## Graphical abstract

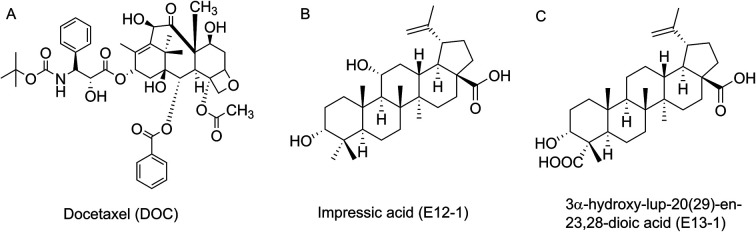

## 1. Introduction

Prostate cancer (PCa) is the most common solid malignancy and the second leading cause of cancer-related death in males.^1^ In the United States, death rates from PCa have exceeded those for lung cancer, making it the most common non-cutaneous malignancy among males.^2,3^ According to the American Cancer Association, in 2015, there were 220 800 diagnoses, accounting for 28% of all male malignancies, and there were 27 540 deaths.^4^ In China, the PCa incidence is lower than that in Western countries but has rapidly increased in the last decade. In addition, the age at onset is decreasing due to lifestyle, longer life expectancy, aging population, and improvements in diagnostic techniques. There are numbers of treatment options available for early-stage PCa; however, 40% of patients will progress to metastatic disease.^5–7^ Many researchers have tried to find effective drugs for PCa from drug synthesis or natural product separation.^8,9^

**Fig. 1:**
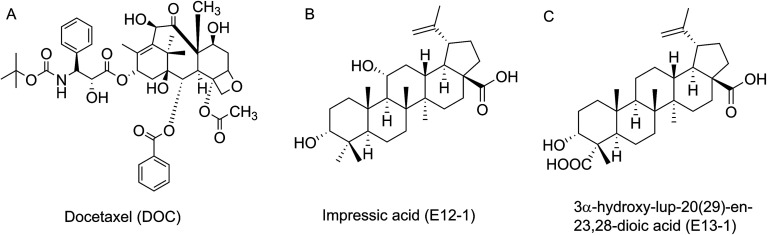
Chemical structures of DOC, E12-1 and E13-1.

The common treatment for patients with PCa is Docetaxel (DOC, Fig. 1A)-based chemotherapy, which has been demonstrated to improve survival.^10,11^ In addition, DOC-based chemotherapy has also become the standard treatment for patients with advanced metastatic PCa who do not respond to androgen deprivation therapy.^12,13^ However, treatment with high doses of DOC ultimately results in resistance and toxicity, limiting the options for patients who experience disease progression with this drug.^14^ Recently, many novel agents such as cabazitaxel, abiraterone, and enzalutamide were developed for patients with PCa following DOC resistance or failure, but their clinical efficiency is limited. Hence, it is urgent that efficacious DOC-based regimens be developed that cause decreased resistance and toxicity.^15,16^

*Acanthopanax trifoliatus* (L.) Merr., a shrub belonging to the Araliaceae family, is widely distributed in Asia including China, Japan, Vietnam, the Philippines, Thailand, and Korea.^17^ It has been utilized as a folk vegetable and medicinal plant for bruising, neuralgia, rheumatic symptoms, and impotence.^18^ In addition, extracts from *A. trifoliatus* (L.) Merr. have several beneficial biological activities, including anti-oxidant, anti-inflammatory, and cytotoxic effects in cancer cells.^19^ Our research group previously showed that crude extracts of *A. trifoliatus* (L.) Merr. have anti-cancer activities in PCa cells. We subsequently isolated 15 compounds from its crude extracts and among them obtained impressic acid (E12-1, Fig. 1B) and acankoreanogein (E13-1, Fig. 1C) which exhibited potent cytotoxic effects against PCa cells.^20–22^ Based on these results, we hypothesized that a combination of E12-1 or E13-1 with DOC would have better effects on the growth and apoptosis of VCaP cells than either compound alone.

In this study, we investigated the potential synergistic effects of E12-1 or E13-1 in combination with DOC on the growth and apoptosis of VCaP PCa cells and the potential underlying mechanisms of their action.

## 2. Results

### 2.1 Inhibitory effects of E12-1, E13-1 and DOC on the growth of VCaP cells

The inhibitory effects of E12-1, E13-1, and DOC on the growth of VCaP cells were evaluated by MTT assays. As shown in Table 1, the IC_50_ of E12-1, E13-1, and DOC were 80.16 μM, 61.17 μM, and 15.71 nM, respectively.

**Table 1:** Inhibitory effects of E12-1, E13-1 and DOC on VCaP cells^a^

Compound	IC_50_
E12-1	80.16 ± 6.58 μM
E13-1	61.17 ± 3.79 μM
DOC	15.71 ± 1.35 nM

a Data are expressed as the means ± SD (*n* = 3).

### 2.2 Trypan blue assays

**Fig. 2:**
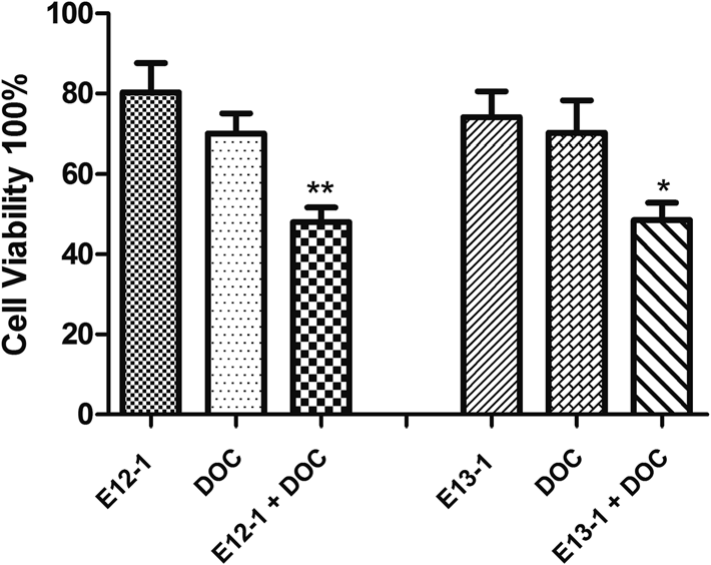
Effects of E12-1, E13-1, and DOC, alone and in combination, on the growth of VCaP cells. VCaP cells were seeded at a density of 0.2 × 10^5^ cells per mL in 6-well plates and incubated for 24 h. The cells were treated with E12-1, E13-1, and DOC alone and in combination for 24 h. Viable cells were determined by the trypan blue assay, and the number of viable cells after treatment with the compounds was expressed as percent of the control. Data are expressed as the means ± SD (*n* = 3) (compared with E13-1 or DOC, *P < 0.05, compared with E12-1 or DOC, **P < 0.001).

The effects of E12-1, E13-1, and DOC alone or in combination on the growth and apoptosis of VCaP cells were determined by the trypan blue exclusion assay. According to the MTT assay, the IC_50_ values, and our previous corresponding studies, we selected 20 μM, 20 μM, and 2 nM as the treatment concentrations for E12-1, E13-1, and DOC, respectively. As shown in Fig. 2, treatment with E12-1, E13-1, and DOC resulted in inhibition of VCaP cell growth inhibition by 19.62%, 25.86%, and 29.10%, respectively. These results indicate that E12-1, E13-1, or DOC alone had little effect on the growth and apoptosis of VCaP cells. However, treatment with a combination of E12-1 + DOC or E13-1 + DOC had significant growth inhibitory effects on VCaP cells. Specifically, treatment with E12-1 + DOC and E13-1 + DOC decreased cell viability by 46.81% and 48.55%, respectively.

### 2.3 Assessment of apoptotic cells by morphology

**Fig. 3:**
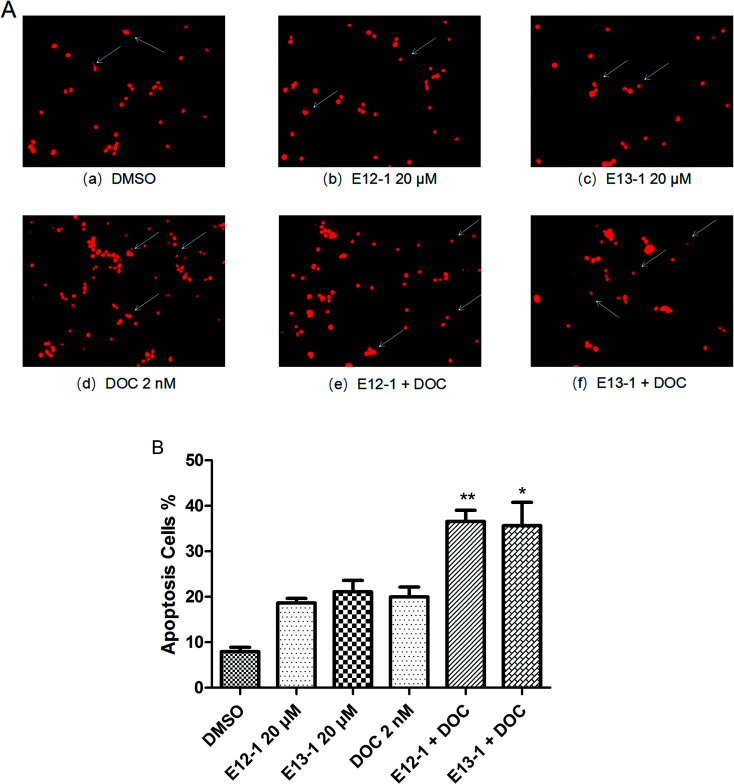
(A) Apoptosis nuclear morphological changes in VCaP cells. Cytospin slides were prepared after the trypan blue assay. Nuclear morphological changes were analyzed by fluorescence microscopy at 200× magnification in cells that had been fixed in acetone/methanol (1 : 1) and stained with PI nuclear fluorescent dye for 10 min at room temperature. (B) Effects of E12-1, E13-1, and DOC alone or in combination on the apoptosis of cultured PCa cells. The arrows indicate the apoptotic cells. Percentage of apoptotic cells after treatment with E12-1 and E13-1 and DOC for 72 h. Each value is the mean ± SD of three separate experiments (compared with E13-1 or DOC, *P < 0.05; compared with E12-1 or DOC, **P < 0.001).

The effects of E12-1, E13-1, and DOC alone or in combination on the apoptosis of VCaP cells were evaluated by morphological assessments. The percent apoptosis with E12-1, E13-1, and DOC alone was only 18.63%, 21.11%, and 19.97%, respectively (Fig. 3B), with slight morphological changes (Fig. 3A[a–d]). However, treatment with E12-1 + DOC or E13-1 + DOC caused visible morphological changes (Fig. 3A[e–f]) compared with E12-1, E13-1, and DOC alone, and the percent of apoptotic cells increased to 36.58% and 35.64%, respectively (Fig. 3B). These results showed that treatment with the combination of E12-1 or E13-1 with DOC strongly promoted the apoptosis of VCaP cells compared with treatment with the compounds alone. The combination index (CI) for 50% apoptosis was calculated to be 0.33 for E12-1 with DOC and 0.41 for E13-1 with DOC, respectively. These results show that the treatment of VCaP cells with a combination of the drugs had stronger inhibitory effects on proliferation and stronger stimulatory effects on apoptosis than the individual drugs alone.

### 2.4 Cell migration

**Fig. 4:**
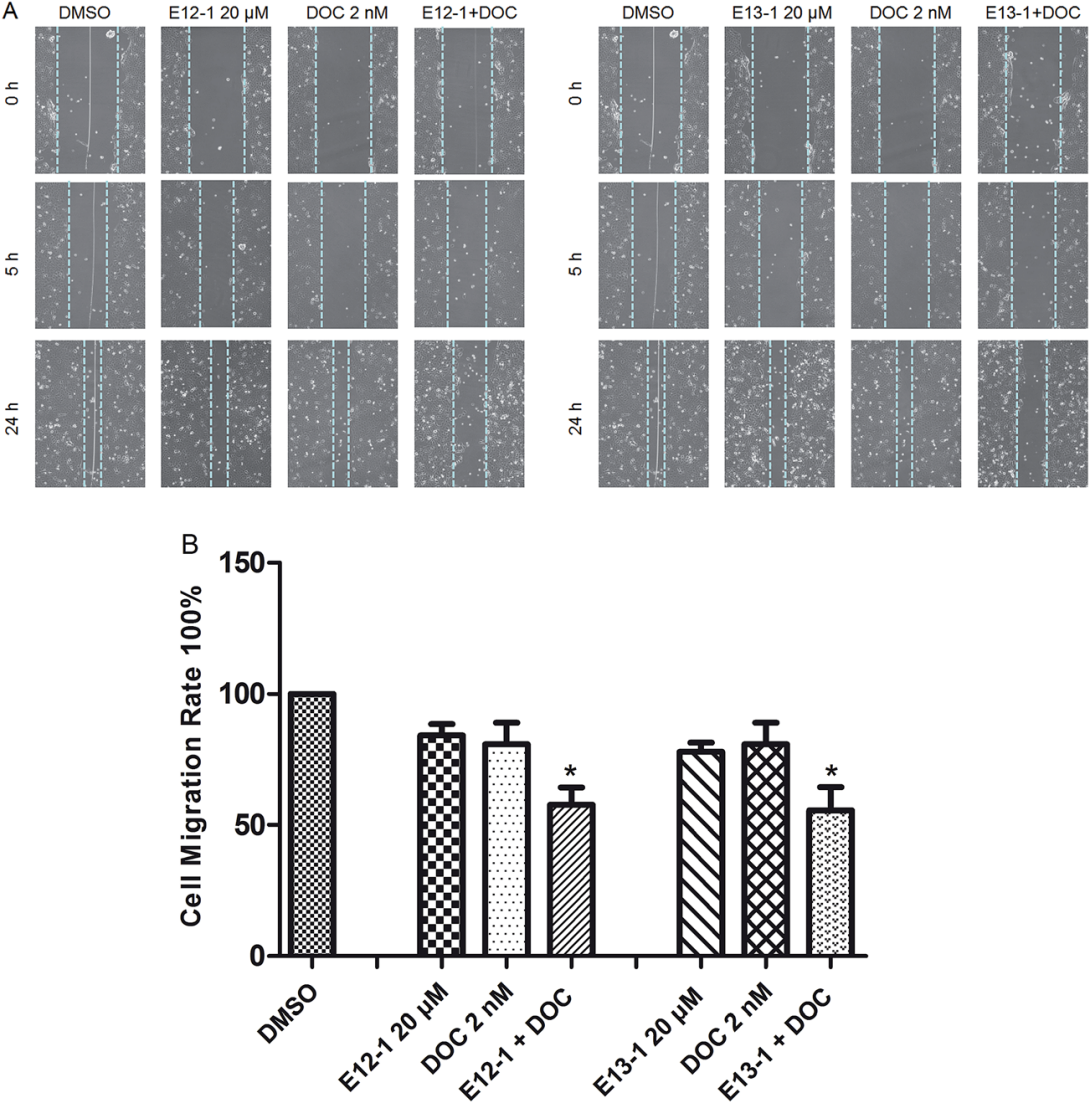
(A) Effects of E12-1, E13-1, and DOC alone or in combination on cell migration as evaluated by the scratch cell migration assay. VCaP cells were seeded at a density of 2 × 10^5^ cells per mL in 6-well plates and the cells were incubated for another 48 h after 90% cytomixis was achieved. The VCaP cells were wounded with a pipette tip and treated with the control, E12-1, E13-1, DOC, E12-1 + DOC, or E13-1 + DOC. Microscopic inspection was carried out by fluorescence microscopy at a 200× magnification after scratching (0 h) and after 5 h and 24 h of cell migration. (B) Percentage of cell-free area at the indicated time points (after scratching for 24 h) compared with that at 0 h was determined. Each condition was tested in triplicate, and the data are expressed as the means ± SD. Experiments were repeated three times (compared with E12-1, E13-1, or DOC, *P < 0.05).

The effects of E12-1, E13-1, and DOC alone or in combination on cell migration were assessed by an *in vitro* cell migration assay. It can be seen from Fig. 4A that after treatment with E12-1, E13-1, and DOC alone or in combination, the cell-free wound gaps healed slowly with time. Compared with E12-1, E13-1, and DOC alone, closure of the wound area with the combination of DOC and E12-1 or E13-1 was significantly decelerated and fewer and fewer cells appeared in the wounded gap, which represented reduce healing of the wound area. E12-1 + DOC and E13-1 + DOC inhibited cell migration by 57.68% and 55.53%, respectively (Fig. 4B), which was significantly lower than the effects with treatment of E12-1, E13-1, and DOC alone, showing that the decrease in cell migration was significantly related to cell proliferation and migration.

### 2.5 NF-κB-dependent reporter gene expression assay

**Fig. 5:**
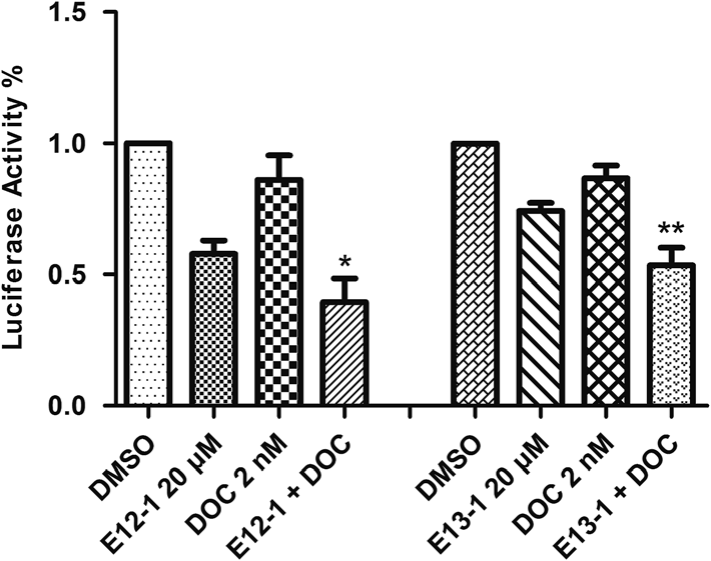
Effects of E12-1, E13-1, and DOC alone and in combination on NF-κB activation in VCaP cells. VCaP cells were seeded in 12-well plates (1.0 mL per well) at a density of 0.25 × 10^5^ cells per mL and then incubated for 24 h. The cells treated with compounds were incubated for 24 h. The NF-κB transcriptional activity was evaluated by a luciferase activity assay. Each value represents the mean ± SD (*n* = 3) (compared with E13-1 or DOC, *P < 0.05; compared with E12-1 or DOC, **P < 0.001).

A luciferase reporter gene expression assay was used to determine the effects of E12-1, E13-1, and DOC alone or in combination on the activation of NF-κB. As shown in Fig. 5, after treatment with E12-1, E13-1, and DOC alone or in combination for 24 h, the NF-κB activity was lower in cells treated with the combination compared with those cells treated with individual drugs.

### 2.6 Effects of E12-1, E13-1, and DOC alone or in combination on the activation of Bcl-2, NF-κB, p-Akt, p-Stat 3, p-JNK, and Erk1/2

**Fig. 6:**
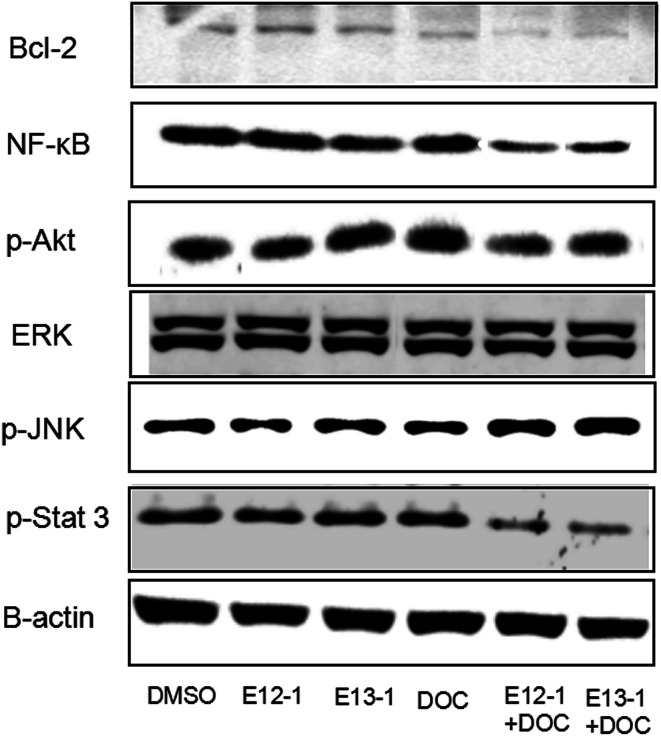
Effects of E12-1, E13-1, and DOC on the levels of Bcl-2, NF-κB, p-Akt, p-Stat 3, p-JNK, and Erk1/2 in VCaP cells. Cells were seeded at a density of 1 × 10^5^ cells per mL of medium in 100 mm culture dishes and incubated for 24 h. The cells were treated with E12-1, E13-1, and DOC alone and in combination for 24 h. Cells lysates were prepared and subjected to western blotting. β-Actin was used as the loading control.

Activation of NF-κB and Stat 3 signaling result in changes in the expression of some target genes involved in apoptosis and cell survival, such as Bcl-2.^23^ JNK plays an important role in insulin resistance, increases the expression of p-JNK, and inhibits expression of p-Akt. The effects of E12-1, E13-1, and DOC alone or in combination on Bcl-2, NF-κB, p-Akt, p-Stat 3, p-JNK, and ERK1/2 were determined by western blot analysis. As shown in Fig. 6, treatment of E12-1 or E13-1 combined with DOC strongly decreased the expression of Bcl-2, NF-κB, p-Akt, and p-Stat 3 and increased the expression of p-JNK compared with treatment with E12-1, E13-1, and DOC alone. The effects of E12-1, E13-1, and DOC alone or in combination on ERK1/2 were small.

## 3. Discussion

Numerous studies have shown that docetaxel, the first-line drug for PCa, presents obvious toxic effects and often results in resistance when used as a high-dose monotherapy. Based on the concept that diversity of cellular targets may decrease adverse effects and overcome drug resistance, there have been many studies on DOC-based treatments in combination with other drugs, but the efficacy of these regimens has remained limited. Our previous study showed that compounds extracted from *A*. *trifoliatus* (L.) Merr. comprising E12-1 and E13-1 had strong inhibitory and apoptotic effects on the growth of VCaP PCa cells.^20^ Thus, the goal of this study was to determine if E12-1 and E13-1, which have low cytotoxicity, could synergistically induce apoptosis and inhibit proliferation in PCa cells when used in combination with DOC.

First, we evaluated the inhibitory effects of E12-1, E13-1, and DOC on the growth of VCaP cells by the MTT assay. The results showed that the IC_50_ of E12-1 was higher than that of E13-1, indicating that E13-1 has a stronger inhibitory effect on the growth of cells. For the trypan blue assay, the results showed that the combination of E12-1 or E13-1 with DOC could clearly promote inhibition and apoptosis compared with either compound alone in VCaP cells. As shown in Fig. 2, the effective growth inhibition with DOC combined with E12-1 or E13-1 increased about 2.5 times compared with either of the compounds alone. Apoptotic cell experiments are one of the most popular methods to assess the toxic effects of drugs. The apoptotic process of cells involves nuclear condensation, intense plasma and nuclear membrane convolution, cell shrinkage and the formation of apoptotic bodies.^24^Fig. 3(A–B) shows that apoptosis in VCaP cells with the combined treatment of E12-1 or E13-1 with DOC significantly increased (*P* < 0.05) compared with that of each drug alone. In particular, E12-1 combined with DOC had the greatest effect.

Cell migration requires structural and molecular changes that allow cells to move from an adhesive state to a migratory state and wounds decrease according to the growth of the cells.^25,26^ Our results (Fig. 4) showed that the combination of DOC with E12-1 or E13-1 showed the highest inhibitory effects on cell migration compared with either of the compounds alone, which is consistent with the cytotoxic activity of the combinations against VCaP cells. The cell migratory effect of DOC combined with E13-1 was better than DOC combined with E12-1.

To determine if the potent effects of combination treatment on VCaP cells were mediated through apoptosis-related signaling pathways, we investigated the transcriptional activity of NF-κB. NF-κB activates genes associated with limitless replicative potential, tissue invasion, and the suppression of apoptosis in PCa cells. Our results (Fig. 5) revealed that treatments of DOC combined with E12-1 or E13-1 decreased NF-κB activity compared with the compounds alone and treatment of DOC combined with E12-1 had higher inhibitory activity than DOC combined with E13-1. Apoptosis occurs through two activation mechanisms, including extrinsic and mitochondrial pathways. The proportion of anti-apoptotic (Bcl-2) to pro-apoptotic proteins is a crucial factor in the regulation of apoptosis because Bcl-2 forms a heterodimer with Bax, inhibiting apoptosis. The NF-κB pathway is linked with the upregulation of Bcl-2.^27,28^ Our results (Fig. 6) demonstrated that overexpression of Bcl-2 occurs in VCaP cells treated with the combination of DOC with E12-1 or E13-1 compared with treatment with either of the compounds alone, which resulted in visible Bcl-2 downregulation.

Akt, Stat 3, JNK, and ERK1/2 signaling cascades are involved in the proliferation, migration, survival, angiogenesis, and lymphangiogenesis of various cancer cells.^29–33^ Thus, we evaluated the effects of the compounds on these signaling cascades and found that treatment of E12-1 or E13-1 combined with DOC caused a strong decrease in the expression of p-Stat 3 and p-Akt and a significant increase in the expression of p-JNK compared with treatment with the individual drugs alone. There were only small effects on ERK1/2 with treatment with E12-1, E13-1, and DOC either alone or in combination.

There is crosstalk between Stat 3 and NF-κB signaling. Stat 3 has oncogenic potential and regulates many aspects of cancer progression, including promoting cell proliferation, promoting cell cycle progression, and inhibiting apoptosis through the induction of genes such as Bcl-2. Our results (Fig. 6) showed that combination compounds strongly reduced p-Stat 3 in VCaP cells. Treatment of DOC combined with E13-1 led to higher inhibitory effects on p-Stat 3 than DOC combined with E12-1, indicating that inhibition of NF-κB and p-Stat 3 activity by the treatment combination was associated with the reduced expression of their target genes. This may a worthwhile direction for future studies.

Akt, the central mediator of the Akt pathway, promotes cell survival by inhibiting pro-apoptotic Bcl-2 family members.^34^ Some PCa cell lines such as VCaP and PC-3 cells express high levels of active Akt. L. Tianyu *et al.*^35^ found that formononetin inhibits cell proliferation and induces cell apoptosis as well as G1 cell cycle arrest by inactivating Akt. In this study, we found that the combination of DOC with E12-1 or E13-1 decreased the expression of p-Akt, while each agent used alone only had very slight effects on p-Akt expression. Treatment of DOC combined with E12-1 had higher inhibitory effects on p-Akt expression than DOC combined with E13-1 (Fig. 6). These results showed that the combination of DOC with E12-1 or E13-1 could effectively inhibit cell proliferation though the downregulation of p-Akt.

Cellular stress and cytokines are the major stimuli that activate the JNK pathway, and mediate apoptosis, proliferation, and cell survival.^36,37^ The ERK1/2 signaling pathway plays an important role in different cellular events and targeting this pathway with synthetic or natural mediators may interfere with other cellular processes, including apoptosis.^38^ Our results showed that the combinations of compounds had stronger stimulatory effects on p-JNK expression and only caused slight decreases on ERK1/2 levels in VCaP cells (Fig. 6). Overall, the results from the trypan blue assay, along with NF-κB luciferase reporter gene expression and western blot analysis, demonstrate that DOC combined with E12-1 or E13-1 effectively inhibits cells growth and induces apoptosis in VCaP cells.

## 4. Experimental

### 4.1 Materials

RPMI-1640 tissue culture medium, penicillin–streptomycin, l-glutamine, and fetal bovine serum (FBS) were purchased from Gibco (Grand Island, NY, USA). Matrigel was obtained from BD Biosciences (Bedford, MA, USA). 3-(4,5-Dimethylthiazol-2-yl)-2,5-diphenyltetrazolium bromide (MTT) and propidium iodide (PI) were purchased from Sigma-Aldrich (St. Louis, MO, USA). Trypan blue stain was obtained from Cambrex (Walkersville, MD, USA). The following antibodies were used: β-actin, phosphorylated signal transducer and activator of transcription 3 (p-Stat 3), and phosphorylated c-Jun N-terminal kinase (p-JNK). They were purchased from Millipore Corporation (Billerica, MA, USA); extracellular signal-related protein kinases 1 and 2 (ERK1/2), phosphorylated Akt (p-Akt), and nuclear factor kappa-light-chain-enhancer of activated B cells (NF-κB) were obtained from Cell Signaling Technology (Beverly, MA, USA); and B-cell lymphoma 2 (Bcl-2) was purchased from Santa Cruz Biotechnology (Dallas, TX, USA). Secondary antibodies were purchased from Santa Cruz. VCaP cells were obtained from the American Type Culture Collection (Rockville, MD, USA).

### 4.2 Cell culture

VCaP cells were maintained in RPMI-1640 culture medium containing 10% FBS, streptomycin (100 μg mL^−1^), penicillin (100 U mL^−1^), l-glutamine (300 μg mL^−1^), and sodium bicarbonate (2.0 g L^−1^). Cultured cells were grown at 37 °C in a humidified atmosphere of 5% CO_2_. E12-1, E13-1, and DOC were dissolved in dimethyl sulfoxide (DMSO), and the final concentration of DMSO in the cell culture medium was 0.1%.

### 4.3 MTT assay

VCaP cells were seeded at a density of 1 × 10^5^ cells per mL in 96-well plates (100 μL per well) and incubated at 37 °C for 24 h. Then the cells were treated with E12-1, E13-1, DOC, E12-1 + DOC, and E13-1 + DOC for 72 h. After treatment, 100 μL MTT solution (10% in colorless RPMI-1640 medium) was added to each well followed by incubation at 37 °C for 1 h. Then the medium was carefully removed and 100 μL of DMSO were added to each well. The absorbance at 570 nm was recorded on a microplate reader. This experiment was done in triplicate for each concentration of the compounds. The effect of different compounds on growth was assessed as percent cell growth compared with DMSO-treated cells. IC_50_ values were calculated as the minimum concentration of each sample required to inhibit cell growth by 50%.

### 4.4 Trypan blue assays

The cytotoxic effects of the combination treatment on VCaP cells were evaluated by the trypan blue assay. VCaP cells were seeded in 6-well plates at a concentration of 0.2 × 10^5^ cells per mL and incubated at 37 °C for 24 h. Then the cells were treated with E12-1, E13-1, DOC, E12-1 + DOC, and E13-1 + DOC for 72 h. After treatment, cells were collected and re-suspended in RPMI-1640 medium. Then the viable cells were measured with a hemocytometer under a light microscope (Nikon Optiphot, Tokyo, Japan). Cell viability was assessed by the trypan blue exclusion assay. Briefly, 80 μL of cell suspension were mixed with 20 μL of 0.4% trypan blue stain solution for 2 min. Blue cells were counted as dead cells, and cells that did not absorb dye were counted as viable cells.^22^

### 4.5 Assessment of apoptotic cells by morphology

Apoptosis was determined by morphological assessment in cells stained with PI. Briefly, cytospin slides were prepared after each trypan blue exclusion experiment, cells were fixed in acetone/methanol (1 : 1, v/v) for 10 min at room temperature followed by 10 min of PI staining (1 μg mL^−1^ in PBS), and then analyzed with a fluorescence microscope (Nikon Eclipse TE200). Apoptotic cells were identified by classic morphological features, including nuclear condensation, cell shrinkage, and formation of apoptotic bodies. At least 200 cells were counted in each sample and the percentage of apoptotic cells was determined.

### 4.6 Cell migration

A wound line was made across all wells with a 10 μL standard pipette tip while the density of the cytomixis was about 90% in each well. Then the wound was washed twice with phosphate-buffered saline (PBS) to remove the cell debris and replace the RPMI-1640 serum-free medium, followed by incubation with E12-1, E13-1, DOC, E12-1 + DOC, and E13-1 + DOC. The cell-free wound area was recorded at the indicated time points of 0, 5, and 24 h using a hemocytometer under a light microscope. Subsequently, the wound area in each picture was determined by outlining the wound and measuring the area using Image J analysis software. From the wound area, the average wound width could be obtained by dividing the area by the length of the analyzed region. The cell migration effect was calculated as the percentage of the remaining cell-free area compared with the area of the initial wound.^39–41^

### 4.7 NF-κB-dependent reporter gene expression assay

NF-κB transcriptional activity was measured using the NF-κB luciferase reporter gene expression assay.^42^ Briefly, VCaP cells were seeded at a density of 0.5 × 10^5^ cells per mL and incubated for 24 h. Then the cells were treated with E12-1, E13-1, DOC, E12-1 + DOC, and E13-1 + DOC for 24 h. Next, the cells were washed with ice-cold PBS and harvested in 1× reporter lysis buffer. After centrifugation, 5 μL aliquots of the supernatants were measured for luciferase activity with a luminometer. Luciferase activity was normalized against known protein concentrations and expressed as a percentage of luciferase activity in the control cells. Protein concentrations were determined with a Bio-Rad protein assay kit (Bio-Rad, Hercules, CA, USA) according to the manufacturer's instructions.

### 4.8 Western blotting

VCaP cells were seeded at a density of 1 × 10^5^ cells per mL and incubated at 37 °C for 24 h. Cells were treated with E12-1, E13-1, DOC, E12-1 + DOC, and E13-1 + DOC for 24 h. Then the cells were rinsed with ice-cold PBS and extracted with 200 μL RIPA buffer (20 mM Tris–HCL [pH 7.5], 1% Triton, 150 mM NaCl, 1 mM EDTA, 1 mM EGTA, 1 mM Na_3_VO_4_, 2.5 mM Na pyrophosphate, 1 mM beta-glycerophosphate, 1 μg mL^−1^ leupeptin, and 1 mM PMSF). The cells were scraped, lysate was collected in a microcentrifuge tube, and cell aggregates were broken up using a 25G × 5/8 needle. Homogenates were centrifuged at 12000 × *g* at 4 °C for 20 min and the supernatant (total cell lysate) was stored at −80 °C. The protein concentrations of whole cell lysates were determined using a Bio-Rad protein assay kit (Bio-Rad). For western blot analysis, 30 μg protein was resolved on 10% SDS-PAGE gels at 100 V for 110 min and electrophoretically transferred to a polyvinylidene fluoride membrane using a semi-dry transfer system for protein detection. Membranes were blocked in 5% fat-free milk in Tris-buffered saline Tween 20 (TBST, 20 nM Tris, pH 7.6, 1.3 mM NaCl, and 0.1% Tween 20) for 1 h at room temperature. Then, the membrane was incubated overnight at 4 °C with the following primary antibodies: p-Stat 3, p-JNK, ERK1/2, p-Akt, NF-κB, and Bcl-2. Following removal of the primary antibodies, the membrane was washed three times with TBST at room temperature, and the blots were incubated with a 1 : 5000 dilution of anti-rabbit or anti-mouse secondary antibody for 1 h at room temperature. After the membrane was washed, enhanced chemiluminescence reagents were added to the membrane for chemiluminescence detection. The membrane pictures were taken on a Bio-Rad Chemi DocTM XRS Imager. β-Actin was used as an internal control to verify equal loading and the band intensities of the proteins of interest were normalized to the internal control. The analysis was performed at least three times.

### 4.9 Statistical analyses

The potential synergistic effects of DOC and E12-1 or E13-1 were assessed using the isobole method with the equation *A*_c_/*A*_e_ + *B*_c_/*B*_e_ = combination index (CI). *A*_c_ and *B*_c_ represent the concentrations of drug A and drug B used in the combination, respectively, and *A*_e_ and *B*_e_ represent the concentration of drugs A and B that produced the same magnitude of effect when administered alone.^43^ If CI was <1, the drugs were considered to act synergistically. If CI was >1 or =1, the drugs were considered to act in an antagonistic or additive manner, respectively. Comparisons of cell viability, apoptosis, migration, and growth inhibition and inhibition of NF-κB activity were analyzed using analysis of variance with Tukey–Kramer multiple comparison tests.

## 5. Conclusion

In conclusion, the results of this study demonstrated that DOC combined with E12-1 or E13-1 markedly inhibited the growth of, and induced apoptosis in, VCaP PCa cells. The effects of the combination treatment were associated with the inhibition of NF-κB activation and the downregulation of Bcl-2, p-Stat 3, p-JNK, and p-Akt. The inhibitory effects of DOC combined with E12-1 were stronger than those of DOC combined with E13-1, whereas the effects on cell migration and p-Stat 3 were better when DOC was combined with E13-1. These differences are due to the different chemical structures of E12-1 and E13-1. Our study provides a foundation for clinical trials to test the combination of DOC and E12-1 or E13-1 as a potential adjuvant therapy for PCa in high-risk populations.

## Ethical approval

This article does not contain any studies with human participants or animals performed by any of the authors.

## Conflicts of interest

All authors declare that they have no conflict of interest in this article.

## Supplementary Material

RA-008-C7RA11647K-s001
