# Supplementary material for: Synergistic effects and mechanisms of impressic acid or acankoreanogein in combination with docetaxel on prostate cancer
Source: RSC Adv. 2018 Jan 12;8(5):2768–76. doi: 10.1039/c7ra11647k (PMC9077455; doi:10.1039/c7ra11647k)
Supplement: RA-008-C7RA11647K-s001 [file RA-008-C7RA11647K-s001.pdf]

Electronic Supplementary Material (ESI) for RSC Advances.
This journal is © The Royal Society of Chemistry 2017

Supporting Information

Extraction and isolation

The air-dried and pulverized stems and leaves of *A. trifoliatus* (10 kg) were extracted with 95% ethanol (50L, 7 days each) at room temperature to yield the extract for three times. The filtrate was combined and evaporated under vacuum and yielded 1380g of crude extract. The crude extract was dissolved in water and extracted successively with Petroleum Ether and an aqueous phase. The aqueous phase was further extracted successively with EtOAc and *n*-BuOH.

The EtOAc fraction (250 g) was subjected to column chromatography (CC) over silica gel eluting with the Petroleum Ether/ EtOc gradient system (40:1, 30:1, 20:1, 10:1, 5:1, 2:1, 1:1, 0:1) and $CHCl_3$/MeOH (5:1, 3:1, 2:1, 1:1) gradient system to afford 20 fractions by using TLC analysis.

Fraction 12 was subjected to a Sephadex LH-20 column ($CHCl_3$–MeOH 1:1) as an eluent and followed by subjecting to CC over silica gel using Petroleum Ether/ EtOc gradient system (80:1) eluent to yield compound **E12** (**1.5** g). Fraction 13 was subjected to a Sephadex LH-20 column using ($CHCl_3$–MeOH 1:1) as an eluent and followed by subjecting to CC over silica gel using Petroleum Ether/ EtOc gradient system (50:1-60:1) as an eluent and puried by CC over Sephadex LH-20 column ($CHCl_3$–MeOH 1:1) to obtain compound **E13** (**1.1** g). By comparison of their NMR data to those reported in the literature, the structures of these compounds were identified as impressic acid[1] and 3α-hydroxy-lup-20(29)-en-23,28-dioic acid[2].

Impressic acid (E12) (Figure 1-2)

White crystal, 1H-NMR (400 MHz, CD3OD),δ:0.84 (3H, s, H-23), 0.94 (3H, s, H-24), 0.98 (3H, s, H-25), 1.07 (6H, s, H-26 and 27), 1.21 (1H, dd, J = 10.5, 7.5 Hz, H-11), 1.52–1.24 (m, 10H), 1.55 (1H, d, J = 10.8 Hz, H-5), 1.68 (1H, t, J = 11.3 Hz, H-18), 1.74 (3H, s, H-30), 2.19–1.85 (4H, m, H2-6, H-15β, H-20), 2.40–2.14 (2H, m, H-15□ and H-16β), 2.65–2.34 (1H, m, H-13), 3.07 (1H, td, J = 10.7, 4.6 Hz, H-19), 3.34 – 3.21 (1H, m, H-3β), 3.87 (1H, td, J = 10.8, 5.3 Hz, H-11β)), 4.64(1H, s, H-29a), 4.78 (brs, 1H, H-29b) 13C-NMR (100 MHz, CD3OD), δ:35.5(t, C-1), 25.5 (t, C-2), 75.7 (d, C-3), 38.8 (s, C-4), 50.0 (d, C-5), 19.2 (t, C-6), 35.4 (t, C-7), 43.4 (s, C-8), 55.4 (d, C-9), 40.3 (s, C-10), 71.1 (d, C-11), 38.5 (t, C-12), 38.3 (d, C-13), 43.8 (s, C-14), 30.7 (t, C-15), 33.4 (t, C-16), 57.4 (s, C-17), 49.8 (d, C-18), 48.3 (d, C-19), 151.5 (s, C-20), 31.8 (t, C-21), 38.0 (t, C-22), 29.5 (q, C-23), 22.9 (q, C-24), 16.9 (q, C-25), 17.7 (q, C-26), 14.9 (q, C-27), 179.9 (s, C-28), 110.5 (t, C-29), 19.6 (q, C-30).

3α-hydroxy-lup-20(29)-en-23,28-dioic acid (E13) (Figure 3-4)

White crystal, 1H-NMR (400 MHz, CD3OD),δ:0.93 (3H, s, H-27), 1.00 (3H, s, H-25), 1.08 (3H, s, H-26), 1.17 (3H, s, H-24), 1.26-1.77 (10H, m)1.72 (3H s, H-30), 1.91-1.94 (4H, m, H2-6, H-15β, H-20), 2.50–2.12 (1H, m, H-13), 3.00-3.08 (1H, m, H-3β), 3.37 (1H, s H-19), 3.73 (1H, brs, H-3), 4.61 (1H, brs, H-29a), 4.73 (1H, s, H-29b). 13C-NMR (100 MHz, CD3OD) δ: 33.5(t, C-1), 26.9 (t, C-2), 73.7 (d, C-3), 52.4 (s, C-4), 45.6 (d, C-5), 22.3 (t, C-6), 35.2 (t, C-7), 42.5 (s, C-8), 51.9 (d, C-9), 38.1 (s, C-10), 21.9 (t, C-11), 26.1 (t, C-12), 39.7 (d, C-13), 43.8 (s, C-14), 30.8 (t, C-15), 33.4 (t, C-16), 57.5 (s, C-17), 50.5 (d, C-18), 47.1 (d, C-19), 152.0 (s, C-20), 31.7 (t, C-21), 38.1 (t, C-22), 180.2 (s, C-23), 17.8 (q, C-24), 16.9 (q, C-25), 16.8 (q, C-26), 15.2 (q,

C-27), 180.0 (s, C-28), 110.1 (t, C-29), 19.6 (q, C-30).

Notes and References

1 Kuo, Y.H., J.M. Lo, and Y.F. Chan. Cytotoxic components from the leaves of Schefflera Taiwaniana. *Journal of the Chinese Chemical Society*, 2002, **49**, 427–431.

2 Chang, S.Y., C.S. Yook, and T. Nohara. 1998. Two new lupanetriterpene glycosides from leaves of Acanthopanax koreanum. *Chemical & Pharmaceutical Bulletin*, 1998, **46**, 163–165.

Figures

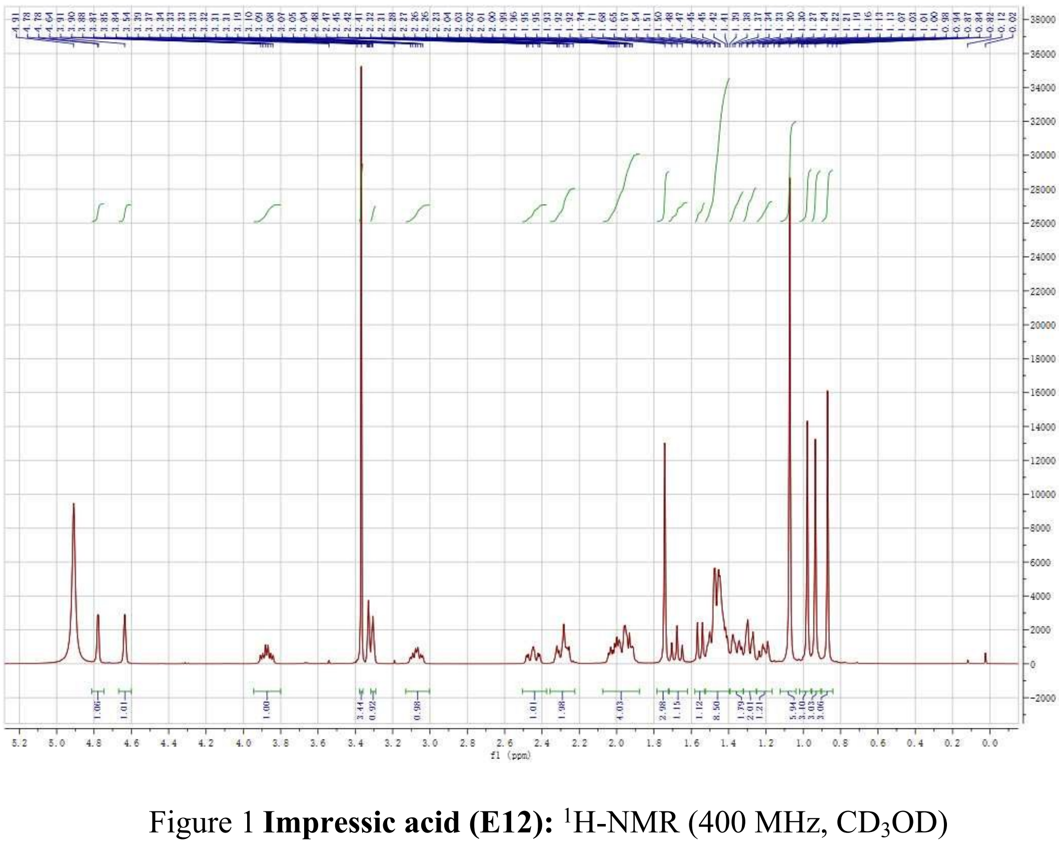

Figure 1 **Impressic acid (E12):** ^{1}H-NMR (400 MHz, CD_3OD)

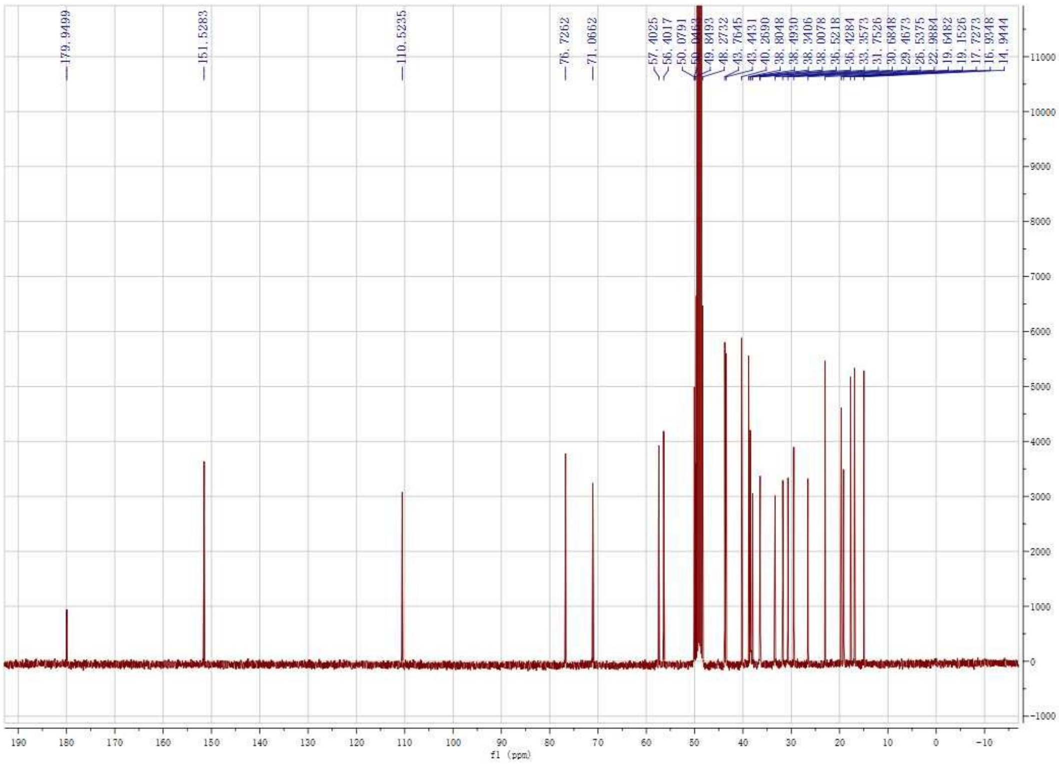

Figure 2 **Impressic acid (E12):** ^{13}C-NMR (100 MHz, CD_3OD)

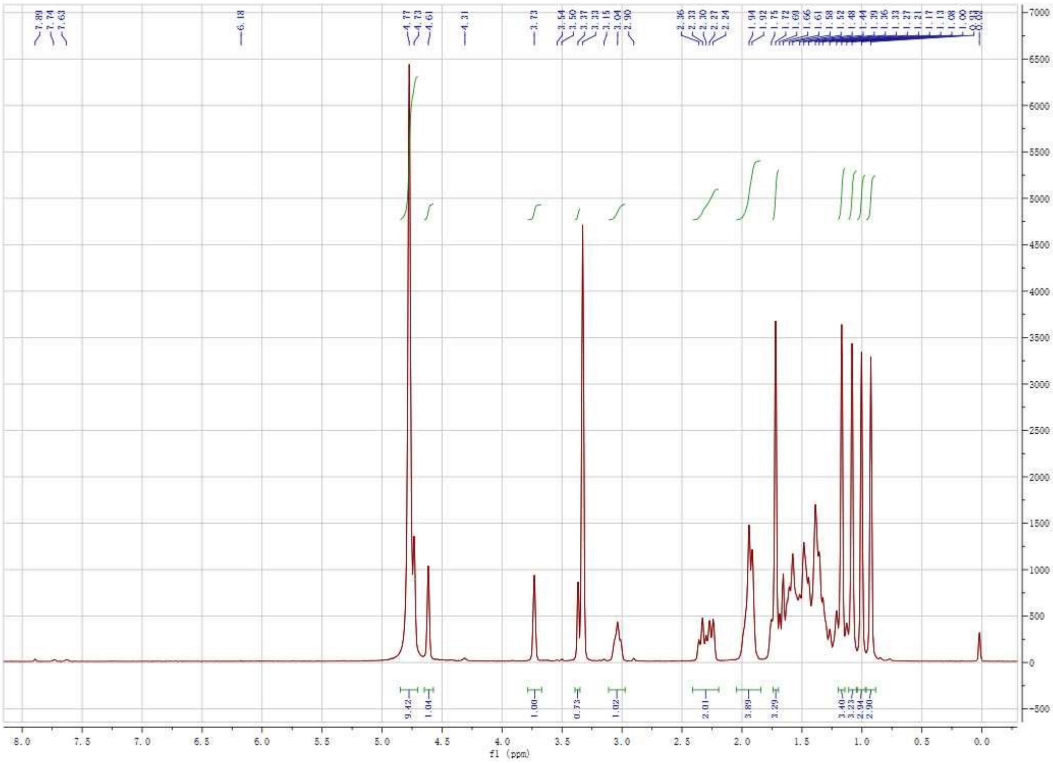

Figure 3 **3α-hydroxy-lup-20(29)-en-23,28-dioic acid:** ^{1}H-NMR (400 MHz, CD_3OD)

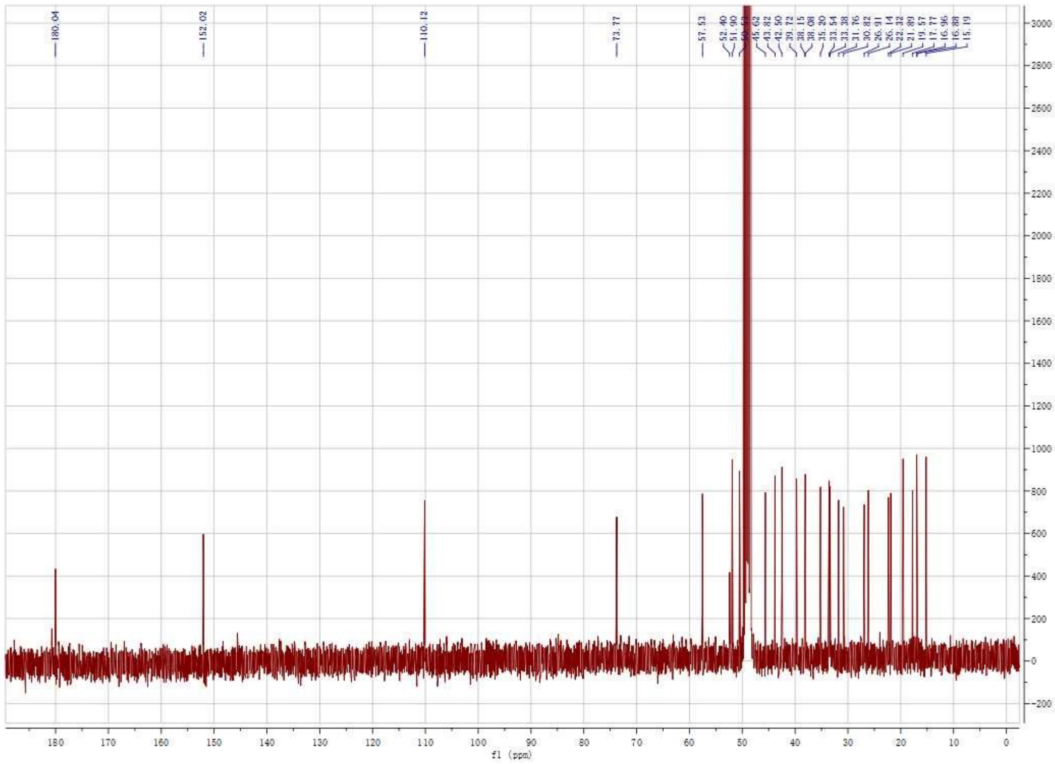

Figure 4 **3α-hydroxy-lup-20(29)-en-23,28-dioic acid:** ^{13}C-NMR (100 MHz, CD_3OD)
